# Public engagement with science: an inclusive approach to innovate in health research with real-world data

**DOI:** 10.1186/s12874-025-02530-4

**Published:** 2025-04-04

**Authors:** Adalton dos Anjos Fonseca, Valentina Martufi, Walisson Angélico de Araújo, Denise Moraes Pimenta, Acácia Mayra Pereira de Lima, Juliana Araújo Prata de Faria, Danilo Luis Cerqueira Dias, Eduarda Ferreira dos Anjos, Maria del Pillar Flores Quispe, Gisela Rodrigues Piloto, Vivian Mitiko Queiroz Lima, Felipe Ferré, Marcos Antônio Gêmeos Almeida Sampaio, Erika de Souza Lopes, Blanda Helena de Mello, Diego Cavalcante Teixeira Daltro, Mariana Rodrigues Sebastião de Almeida, Raiza Tourinho Lima, Elzo Pereira Pinto Junior, Mauricio L. Barreto, Maria Yury T. Ichihara

**Affiliations:** 1 Centre for Data and Knowledge Integration for Health (CIDACS), Gonçalo Moniz Institute, Oswaldo Cruz Foundation (FIOCRUZ), Salvador, Brazil; 2 State University of Southwest Bahia (UESB), Salvador, Brazil; 3 Federal University of Sao Paulo, Federal University of Sao Paulo, Sao Paulo, Brazil; 4 Federal University of Paraná (UFPR), Federal University of Paraná (UFPR), Curitiba, Brazil; 5 Municipal Health Secretary of Salvador, Salvador, Brazil; 6 National Council of State Health Secretaries (Conass), Brasília, Brazil; 7 State Health Council of Bahia, Salvador, Brazil; 8 Umane, São Paulo, Brazil; 9 Ministry of Health, Brasília, Brazil; 10 State Health Secretary of Bahia, Salvador, Brazil

**Keywords:** Public Engagement with Science, Real-World Data, Primary Health Care, Data Interoperability, Health Data

## Abstract

**Background:**

Public engagement with science (PES) initiatives in health research that use big data to analyze social inequalities in health requires strategies and methods adapted to the contexts of countries in the Global South. This paper aims to examine how, in Brazil – a country with a strong tradition of social participation in research and public policymaking—two research projects from a center that utilizes administrative and real-world data incorporate inclusion and diversity as key elements to connect knowledge production with real-world challenges.

**Methods:**

The study analyzes how two Cidacs research projects – one related to Primary Health Care (PHC) and the other to Data Interoperability—involved members of the public throughout their implementation. Both projects jointly engaged 18 participants, including community representatives, health professionals, and public sector managers. A case report approach is being employed in this paper to systematically document PES experiences based on a predefined script, covering context, methodology, activities, audiences, and ethical aspects. Data were collected through participant observation and listening during engagement activities, which facilitated dialogue between participants and researchers, as well as through follow-up questionnaires and subsequent discussions. This paper itself emerged from this collaborative process, including with some PES participants as co-authors.

**Results:**

The participants' collaboration impacted the researchers' decisions, providing a closer understanding of the challenges faced by the participants in their daily work in relation to each of the research themes. Furthermore, these discussions resulted in the establishment of partnerships for new initiatives. The participants highlighted that, in addition to the opportunity to contribute to the development of scientific research, they acquired new knowledge from their contact with the research teams.

**Conclusions:**

The diversity of social groups and the inclusion of different perspectives in research projects mobilized by PES have the potential to promote innovations in research processes and results, as well as have social impact. The potential for applicability of scientific information is expanded since it is more connected to the real world, and the participants themselves drive the dissemination process.

**Supplementary Information:**

The online version contains supplementary material available at 10.1186/s12874-025-02530-4.

## Context

Publications on public engagement in science (PES) in health research have grown significantly. On the PubMed platform, the number of articles increased from 77 to 258 between 2013 and 2023. 1The initiatives range from actions in which participants influence health decisions [1, 2], suggesting improvements in health services [3] or participating in research that applies participatory methodologies, as in Rubio et al. [4]

PES refers to one of the levels of participation of different social segments in the process of constructing scientific knowledge [5]. Initially developed in countries of the Global North, it is better known in Brazil as social participation. It is deeply linked to the struggles for human rights and is in alignment with social movements. A good example of this has been the creation and building the Brazilian Unified Health System (SUS). SUS was created in 1988 and aimed to reduce health inequities in the country by guaranteeing universal, comprehensive, and free access to health services for all [6, 7].

Some of the actors who participated in the social struggles for establishing the SUS, from academia and public management, founded the Center for Data and Knowledge Integration for Health (Cidacs/Fiocruz Bahia) in 2016. The research center was created to carry out multidisciplinary studies that use the linking of large administrative databases to generate evidence on the social determinants of health and to assess the impact of public policies on the health of the population, using Real-World Data (RWD) [8]. An outstanding achievement of the Cidacs was the creation of two very large cohorts using exclusively linked administrative databases [9, 10]^.^

This article presents a methodological approach to PES based on strategies to enable inclusion and diversity in health research carried out with RWD. Using two experiences of Cidacs research projects related to Primary Health Care (Cidacs PHC) and data interoperability (Cidacs-PHDC), we will present the challenges and solutions developed, seeking to contribute to the systematization of a strategy in research processes that promote inclusion and diversity in knowledge production. We will also demonstrate the impacts and lessons learned from these initiatives.

## Defining public engagement with science

Public engagement with science (PES) refers to the inclusion, in the knowledge production process, of voices commonly marginalized in public debates and not directly involved in professional activities related to scientific disciplines [5, 11, 12]. It also extends to policymakers, professional communities, legislators, NGOs, and intergovernmental institutions' participation as a strategy to consult, collaborate, and co-create useful knowledge that informs collective decision-making [1].

The World Medical Association's Declaration of Helsinki [13] emphasizes the importance of involving participants and their communities before, during, and after research. Meanwhile, Arnstein [14] highlights the need for a redistribution of power throughout the participation process, advocating for a fundamental restructuring of decision-making dynamics and the relationship between science and society. The Spectrum of Public Participation is a useful model from The International Association of Public Participation (IAP2) that categorizes the levels of citizen involvement in decision-making based on five modes of participation – inform, consult, involve, collaborate and empower.

Researchers in the United Kingdom and the United States shaped this concept within the scientific community. However, over time, it has been embraced on a wider scale [5, 12]. In Latin America, various adaptations have been implemented due to the region's unique social, political, and cultural contexts, and its strong tradition of social participation. Revolutionary initiatives by Brazilian activists in popular education and public health, such as those of Paulo Freire [15, 16] and Sergio Arouca [17], have been fundamental for the construction of a society widely engaged in the fight for rights. The initiatives in the region are guided by diverse principles and values. Nontheless, empowerment is a central theme, with community groups actively influencing health decisions and gaining the capacity to make informed choices. Diversity is important to ensure a variety of viewpoints. Inclusion and collaboration among researchers, health professionals, policymakers, and communities are emphasized in these activities that address real-world challenges [18, 19].

At Cidacs, PES focuses on sharing experiences and knowledge between researchers and stakeholders of the health system to generate evidence that supports social policies. The work done goes beyond community groups – e.g. traditional populations or favela residents—since there are collaborations with public managers, policymakers and intergovernmental organizations (such as the World Health Organization and the United Nations), advocacy groups and civil entities, health workers, media and education professionals, as well as academics specialized in the topics studied. The involvement of these stakeholders is carefully planned and progressively implemented, considering their specific demands and needs. The results of this collaboration have been significant for research projects, making them more aligned with the real world and increasing their potential for applicability.

**Fig. 1:**
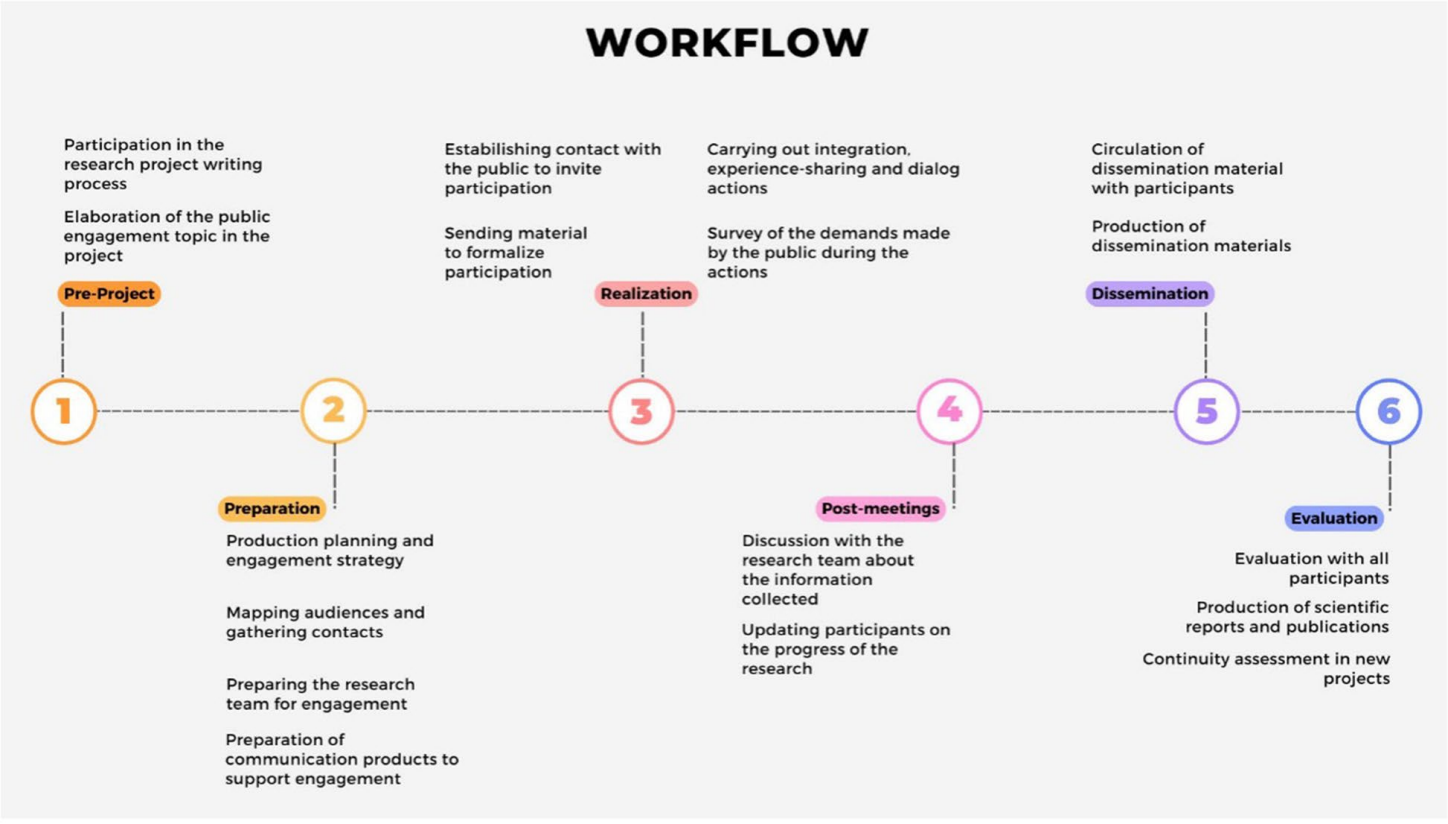
Workflow of public engagement with science activities at Cidacs. Source: authors’ own design

Within the scope of this work, the Cidacs team develops a series of tasks, ranging from research to evaluation, according to the workflow below (Fig. 1).

## Methods

The cases presented involve two research projects carried out at Cidacs, including different audiences – community representatives, health professionals, and health management technicians – from the onset of the research process. Since the conception of each research project, researchers indicated the intention of carrying out PES, adapting guidelines of Sect. 6 of the World Medical Association's 2024 Declaration of Helsinki to the Brazilian context. The procedures for selecting and building relationships with participants in the public engagement activities of both projects followed these key steps:

1. Mapping relevant institutions at local, state, and national levels related to the main topics of the projects;
2. Validating the list of institutions with senior researchers from the project and five stakeholders from other research initiatives at Cidacs through individual conversations;
3. Identifying participants within these institutions to accompany the project;
4. Sending formal invitations explaining the initiative;
5. Establishing a relationship of trust with stakeholders by emailing periodic updates about the projects.

A total of 18 individuals participated in the activities of the two projects, out of 27 individuals invited to join the group. Among them, 11 were public sector managers, four were healthcare professionals, two represented civil society organizations, and one was from the academic community. Their roles ranged from coordinators or advisors in the PHC sector, family doctors, nurses, or health council members. The majority of members were women (13) and representatives of local and state-level institutions (13). Additionally, five participants represented federal-level institutions.

The engagement activities included in-person and online technical meetings where researchers and participants discussed research questions, methodologies, data sources, analytical tools, and result interpretations, as detailed in the following sections of this article. Data were collected through participant observation during the engagement activities and through structured questionnaires and open-ended feedback forms.

To present the initiatives of interest, we adopted the case report – a form of knowledge production based on critical reflection from an academic or professional experience. Based on a script [20] we systematized the inclusive experiences of PES based on their contextualization, the methodological procedures adopted for each intervention, and the characterization of the research projects in terms of time, space, activities, audiences, and ethical aspects.

### PES Initiatives for the CIDACS PHC and CIDACS-PHDC projects

The initiatives reported are based on the same model for structuring the procedures and values ​​involved in PES activities, although some adjustments are made to meet the specific needs of each project. To set up the engagement activities for the projects described below, researchers associated with the projects together with the engagement team – made up of communication and anthropology researchers – held meetings to: 1) Map potential participants, encouraging representativeness according to race, gender, age group, region, and different functions/positions; 2) Develop recruitment strategies (use of institutional emails, instant messaging, telephone, and social media to send invitations with a message about the project and the workshop, as well as the gradual sharing of complementary/informative materials about the project); 3) Organize workshop scripts using accessible language, including guiding questions, and train the research team to give space and effectively listen to the experiences of the stakeholders; 4) Develop an evaluation model for the activities and results obtained. All engagement activities are supported by the Cidacs scientific communication and dissemination group, which ensures levels of engagement through consulting, involvement, and collaboration.

#### Cidacs PHC

The Cidacs PHC group is dedicated to studying the impact of Primary Health Care (PHC) on the population's health. In the first phase of the project, the effect of the quality of PHC on child mortality was explored. In its second phase, with a structured PES strategy, the focus has been on deepening findings from the previous phase by including research questions on the determinants of PHC quality. The first engagement activity was a workshop at the Cidacs headquarters in Salvador (Brazil), where fifteen people, including ​​public health managers, health workers, researchers from other institutions and representatives of civil society, discussed the results from the first phase of the project and research questions of the second phase (activity outline in the supplementary material). This first introductory engagement activity was in-person and lasted a full day, which allowed for the extensive coverage of all the research questions for this project. Subsequently, three follow-up meetings were focused on single research questions, held online and with a shorter duration (90 min), due to logistic and budgetary constraints. These represented the continuation of the engagement process, as the research group advanced with the analyses and brought back their findings to the engagement committee for discussion and joint decision making with regards to next steps.

#### Cidacs-PHDC Project

There is a collaboration between Cidacs and the Western Cape Provincial Health Data Centre (PHDC) in South Africa that aims to create a Common Data Model (CDM) to study infectious diseases that affect pregnancy, such as gestational syphilis and tuberculosis, using data from Brazil and the Western Cape Province. The creation of a CDM aims to structure the databases from each institution according to a unified standard of tables, fields, and relationships designed to harmonize data from different sources. To this end, it uses controlled vocabulary, concepts and codes, ensuring a consistent interpretation of data regardless of its origin. An engagement activity with Big Data experts and health managers from national and local government bodies was carried out to discuss the development and application of the CDM in the Brazilian and South African contexts (activity outline in the supplementary material). During the engagement session, participants exchanged experiences and discussed topics related to the utilization of a common data model that considers the social and economic specificities of the Global South. These discussions were fundamental in the development of a CDM that promotes data standardization from an inclusive and collaborative perspective. The decision-making process regarding CDM adoption by governments will influence the availability of good quality data for researchers to conduct meaningful analyses that can inform policymakers and improve health systems. Three representatives from Brazil's public administration and two representatives from the Department of Health of the Western Cape Province of South Africa participated in the first PES activity for this project. The main product of this initiative is a recommendation document about CDM for stakeholders – professionals, policymakers and other institutions.

### Activities with participants

The engagement activities for both projects included a combination of in-person and online meetings, fostering meaningful dialogue between researchers and participants. In the Cidacs-PHC project, participants were invited to critically review, expand, and refine research questions, data sources, analytical approaches, and result interpretations. Researchers’ presentations were followed by focused discussions, where participants could share suggestions, critiques, and questions, creating a collaborative and inclusive environment. To ensure transparency and continued engagement, all presentation materials were shared with participants after the sessions, allowing for further reflection and input.

In the Cidacs-PHDC project, participants and researchers presented solutions and experiences on data interoperability from their respective workplaces, highlighting the diversity of approaches across different contexts. Following the presentations, a discussion forum was established to facilitate the exchange of ideas and potential collaborations. This inclusive approach enabled participants to contribute to the research project's objectives, methodologies, and final outputs, guiding new directions and possibilities for future work.

Additionally, it is important to highlight that the production of this article itself is a result of this inclusive cooperation strategy, as some participants are also co-authors. All participants were invited to contribute through an email containing five open-ended questions, with the option to respond via an attached document or an online form. In total, 18 individuals were invited, and 7 agreed to participate, submitting contributions that reflected their perspectives on the engagement activities and reviewing the manuscript.

## Results

### Engagement process

During the meetings (two in-person and three online), issues were addressed from different perspectives and realities, which resulted in a rich experience of sharing different approaches to solve complex problems. Participants appreciated the opportunity for reciprocal learning and knowledge exchange with professionals from different levels of expertise. They also pointed out that this research model based on the inclusion of ideas from diverse people allows science to be closer to the territory and achieve more relevant, inclusive and representative results.

### Impact on the research projects

For the Cidacs PHC team, the meeting with participants provided five main takeaways: i) the need to urgently include racial issues within the scope of the analyses; ii) the importance of identifying other variables to assess the quality of PHC, such as the type of employment contracts and work overload to represent the quality of the workforce; iii) the potential impact of the professional profile and gender of the Health Secretary on the adequacy of municipal management; iv) the context and nuances related the time frame of the research; and, v) the agreement of important topics, such as the time lag between the reception of financial resources and manifestation of improvements in the quality of PHC.

For the Cidacs-PHDC project, the activity allowed the validation of the importance of this movement to build capacity for data interoperability and the application of a CDM, to promote the use of real-world data. All participants recognized that a specific CDM, developed by the OMOP (Observational Medical Outcomes Partnership) is a valid path, from the perspective of open science, to be explored to break down frontiers in health knowledge.^2^ However, the data managers who participated in the discussions indicated that the OMOP CDM specifically is more helpful for research and production of knowledge on health, whilst not being particularly useful for the management of health data by state entities. In fact, in both countries, other CDMs are already being tested with a focus on the primary use of health data to support service provision.

In this regard, the Digital Health Strategy adopted in Brazil establishes governance for the exchange of information with models agreed upon by the Tripartite Intermanagerial Commission (CIT). Participants noted that reconciliation between academic solutions and management strategies could accelerate knowledge discovery, reduce translational response time and, simultaneously offer timely support for decision-making for citizens, professionals, and managers. They suggested new stakeholders and community groups to include in this discussion, as well as some advocacy strategies.

### Continuous evaluation

The continuous evaluation process, which assesses participants' level of interest and engagement, involves analyzing suggestions, criticisms, and comments shared during and after the meetings. Informal testimonies and plenary assessments also served as valuable sources of insight. Additionally, a post-activity evaluation questionnaire was administered, offering a structured approach to assess participants' satisfaction and confidence in the adopted process. Responses were 100% positive, with the four close-ended questions addressing key aspects such as the overall quality of the meeting, the methodology, the clarity of presentations, the interest in continued participation, the highlights, and suggestions for improvement. However, one challenge emerged in the descriptive responses: the complexity of the project topics often exceeded the time allocated for discussion, which was limited to 40 min to 1 h per topic. Despite this, the sustained involvement of participants in subsequent engagement activities (return rate exceeding 90%), as well as the co-authorship of this manuscript by some participants reinforce the establishment of a relationship built on trust.

## Discussion

The operationalization of key concepts such as diversity and inclusion are complex tasks. The development and implementation of these processes involve discussions mainly around race and gender, which are structural factors in the processes of construction of inequalities in our region [21] but also intersect with other categories such as socioeconomic status, age, sexual orientation, religious beliefs, ideologies, physical abilities, among others. Discussing these issues demands considerations regarding how, throughout history, theories and methodologies from the Global North were imported into the Global South without problematizing their epistemological and techno-scientific impact [22].

Developing a common data model for health research without incorporating the contextual specificities of populations in countries with deep social inequalities highlights a significant challenge. Knowledge production, often driven by the Global North in the pursuit of international data standardization, must actively include diverse perspectives that can only be fully understood through real-world practice. Failing to account for these contextual features risks creating data models misaligned with the realities of vulnerable populations, ultimately limiting their representativity and effectiveness. The outcomes of such an approach will significantly influence how these populations are analyzed and monitored, directly impacting the development of public policies.

The PES activities reported in this paper presented important results due to the efforts to meet the diversity of social groups' profiles and include different perspectives in research projects. In addition to gender and race as inclusion criteria, other important aspects to build a diversity of profiles were considered for the Cidacs PHC project, such as job function, academic background, professional experience, hierarchical level and institution of origin. The Cidacs-PHDC activity, on the other hand, involved a smaller number of participants due to the complexity and specificity of the topic discussed – data interoperability and CDMs. Nonetheless, an effort was made to represent different government institutions for a richer discussion. However, the opportunity was used to explore the mapping of target audiences for greater diversity.

Understanding the regional context, with a focus on inclusive and diverse strategies that promote cooperation between researchers and society, is essential for interventions related to the engagement process [23]. In this sense, some lessons from Reynolds and Sariola [24] permeated our decisions, manifesting in engagement processes such as active listening regarding participants' everyday experiences, the impact of participants' contributions on research processes, and the establishment of new partnerships and collaborations.

This inclusion process also involves some barriers that limit the adopted strategies. Time was one of the main barriers indicated by all participants. Furthermore, the team noted that the inclusion of people from different hierarchical levels in the Cidacs PHC project activity, although important for accessing different points of view, resulted in constraints in the expression of divergent ideas due to power imbalances within the participants. In the case of the Cidacs-PHDC project, the different contexts and languages ​​were also aspects that needed to be noted in the PES process. Also, implementing some of the participants' suggestions was difficult due to the lack of specific variables in the available databases. However, this also represented an opportunity to reflect on new data sources that could be used, as well as enrich the researchers' process of actively acknowledging the limitations of their work. We also recognize the potential for bias in the participants mapping of both projects; however, this approach was chosen primarily due to travel cost considerations and the rapid mobilization of participants.

Lessons learned include the importance of detailed planning of each stage of the engagement work, from the objective and stakeholder mapping to the scheduling of each session, following recommendations by dos Anjos Fonseca [5]. Formulating guiding questions for debate between researchers and participants proved to be very beneficial because it mobilized participants and stimulated discussions.

**Fig. 2:**
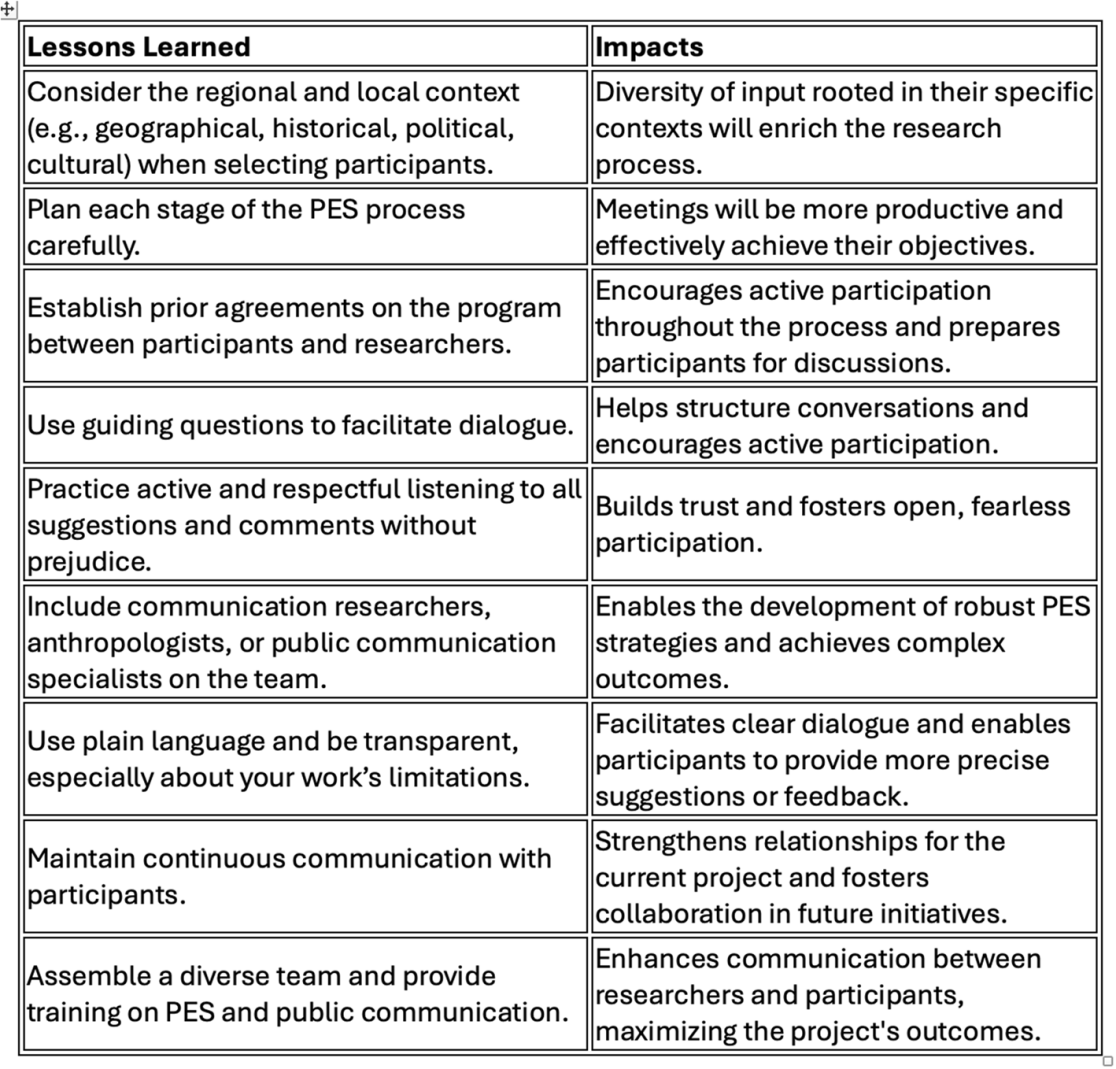
Lessons learned and impacts of PESC in both initiatives. Source: authors’ own design

Another important aspect of this relationship built between researchers and participants is the ethical dimension of public engagement. Critical ethical dimensions that make up the framework for participation processes and citizen science [25] were contemplated, such as empathy and the construction of communicative spaces, the raising of questions that permeate ethical aspects – such as data ethics and issues of race and gender –, the promotion of dialogue and the fostering of relationships without prejudice. In this sense, communication is fundamental for structuring an ethical and collaborative relationship between researchers and PES participants. The inclusion of communication researchers and an anthropologist in the team was important because it emphasized the importance of transparency, raising awareness among researchers about delivering presentations with an accessible language, and promoting active listening and prior agreement on the program between participants and researchers. This was done through meetings, strategic messages sent to participants before and after the activities, informal conversations and evaluation processes during the meetings. Further details on the impact of the PES process on these research projects are detailed in Fig. 2). Figure 2 summarizes the main lessons learned during the Public Engagement with Science experiences.

As this intervention demonstrates in practice, a barrier to overcome is that science should be produced not only for society but with society. By considering diversity and going beyond the academic frontier with this type of intervention in the health area, Cidacs integrates knowledge and subverts the pattern of elites "deciding for" the masses. This movement is also essential for the democratization of knowledge and for increasing trust in research institutions, their production and recommendations. In addition to expanding the understanding of data, PES activities provide additional contextual information that enriches research based on findings from the analysis of extensive health databases, connecting the findings to local realities and the concrete challenges and needs of the population.

The following steps for these initiatives include continuing engagement activities to discuss the future directions of the research projects. This will involve expanding the group overseeing the Cidacs-PHDC project to include representatives of community groups and civil society organizations, ensuring diversity in representation through carefully defined criteria. Additionally, participants will be consulted and involved in shaping strategies for scientific dissemination and advocacy.

## Conclusions

The experiences reported in this manuscript highlight the strategic role of public engagement with science and communication in the execution of processes that help to build and strengthen the articulation of knowledge production with everyday reality. This allows the scientific community to engage in dialogue with various social segments that work directly with the research topics of interest.

Although the projects are still ongoing, it is evident that combining PES, communication, and interdisciplinarity highlights the critical role of quality listening and meaningful dialogue in identifying common ground and fostering innovations that help mitigate health inequalities. Achieving this requires more inclusive research approaches that embrace diverse perspectives and adapt methodologies to address populations' specific needs better. We also expect impacts in influencing data experts, policymakers, and researchers as well as in developing new initiatives that promote diversity in research teams.

Reporting these experiences of cooperation and dialogue, in addition to data analysis and theoretical discussions, allows us to understand the importance of humanizing health services and science. Expanding repertoires through dialogue and listening has proven to be essential for implementing more inclusive practices that go beyond quantitative indicators, valuing specific contexts and promoting meaningful connections.

Recognizing that access and capabilities are unequal – both in science and in health care – is a fundamental step to avoid perpetuating inequalities. Scientific methods often fail to consider the specificities of territories and the populations inhabiting them. We recommend building capacity in public communication of science and PES to apply participatory methodologies in these practices. Additionally, it is important to go beyond data and numbers when conducting population health research. By listening and developing inclusive dialogues and connections with different stakeholders and giving up pre-established certainties to expand one's capacity to welcome other perspectives, it could be possible to implement innovations and contribute to a more equitable future without repeating past mistakes.

## Supplementary Information

Additional file 1. Invitation for coproduction of scientific article & questions sent.

Additional file 2. Agenda for the pes activity—﻿Cidacs-PHC project.

Additional file 3. Agenda for the pes activity—Cidacs-PHDC Project.

Additional file 4.

## Data Availability

No datasets were generated or analysed during the current study.

## Footnotes

1 Search conducted on February 21, 2025

2 The OMOP CDM is supported by the Observational Health Data Sciences and Informatics (OHDSI) community, whose mission is to improve the health of the population by empowering researchers to collaboratively generate evidence that promotes better health decisions and improved care. Although the OMOP CDM is primarily designed around clinical data modeling, Cidacs saw an opportunity to collaborate with the international community by promoting the inclusion of socioeconomic data in studies conducted, thus expanding the applicability of the CDM in the Global South.
