# Supplementary material for: Public engagement with science: an inclusive approach to innovate in health research with real-world data
Source: BMC Med Res Methodol. 2025 Apr 4;25:88. doi: 10.1186/s12874-025-02530-4 (PMC11970009; doi:10.1186/s12874-025-02530-4)
Supplement: Supplementary file 1 — Additional file 1. Invitation for coproduction of scientific article & questions sent. [file 12874_2025_2530_MOESM1_ESM.docx]

**ADDITIONAL FILE 1**

**INVITATION FOR COPRODUCTION OF SCIENTIFIC ARTICLE**

**& QUESTIONS SENT**

Hello,

In recent years, the Center for Data and Knowledge Integration for Health (Cidacs), lab of Fiocruz Bahia, has dedicated itself to developing and promoting public engagement with science (PES). This initiative is directly related to the democratization of scientific research and the relevance that contributions from society have for the quality of science carried out at Cidacs and in Brazil as a whole.

Consequently, most of the research carried out by Cidacs has been aligned with PES, which enables the exchange of knowledge, directly impacting scientific production and results. In other words, the dialogue with the most diverse social groups - for example, managers, community representatives, health workers, etc. - has positively impacted our research.

In this regard, Valentina Martufi and Elzo Pereira Pinto Júnior, coordinators of two important projects at the Center, “A common data model of pregnancy IDs with real-world data from the Global South” and “Impact of Primary Health Care on Maternal and Child Morbidity and Mortality in Brazil”, research projects being developed in alignment with PES, have recently suggested the co-production of a scientific article with the stakeholders who have contributed so much to these investigations.

The purpose of this text is to reflect on the methodology of interaction and engagement of the two projects mentioned above with the people who have collaborated in the research, whether in technical meetings, focus groups, surveys, discussions, etc. Understanding that there are numerous ways to collaborate and author an article, we would like to invite you to contribute to the co-production of this scientific product. The Cidacs team will be responsible for writing the text and the other coauthors will contribute by answering some questions and sending reflections by email, as well as reviewing the manuscript’s first draft. Remember that, regardless of the form of contribution, all those who participate will be authors of the text.

The article will be submitted by 11/30/24 to the journal BMC Medical Research Methodology, which is accepting submissions for the collection "Inclusive Methodological Awareness for Equity and Diversity". This collection seeks to promote innovative research techniques that address health disparities and encourage inclusion and equity. Said submission will be made at no cost to stakeholders and collaborators.

We hope that you are interested in participating. To that end, we have some brief questions that will help in the composition of the article. We thank you in advance for your attention and collaboration. Below are the questions to be answered (the answers can be included in this document, which should be sent as an attachment to this email, or, if you prefer, through the online form (link below):

**What is your name?**

**_________________________________**

**Would you like to participate in the production of this article?**

**( ) Yes ( ) No**

**If you have agreed to participate in the co-production of this article, please answer the questions below:**

If you prefer, you can respond using the electronic form at the following link: <https://forms.gle/34en6EbiEdcTcsSk8>

1. What are your impressions from your experience participating in the project? Tell us a little about the activities you participated in and the impact they had on you.
2. What benefits and challenges do you see in the interaction of social groups with the research project in which you participated?
3. Tell us a little about what you think about society’s engagement with scientific projects like these from Cidacs.
4. Would you like to add anything else?
5. What are the most appropriate day(s) of the week and time to schedule a time to discuss this activity, if necessary?

Observation:

If you prefer to submit your responses in a document rather than using the online form, we suggest saving them in PDF format, which is a more recommended file type to preserve the original formatting.
