# Supplementary material for: Public engagement with science: an inclusive approach to innovate in health research with real-world data
Source: BMC Med Res Methodol. 2025 Apr 4;25:88. doi: 10.1186/s12874-025-02530-4 (PMC11970009; doi:10.1186/s12874-025-02530-4)
Supplement: Supplementary file 2 — Additional file 2. Agenda for the pes activity—﻿Cidacs-PHC project. [file 12874_2025_2530_MOESM2_ESM.docx]

**ADDITIONAL FILE 2**

**AGENDA FOR THE PES ACTIVITY - CIDACS PHC PROJECT**

**Construction Workshop for the 2nd phase of the project being developed by the Research Group on Primary Health Care and Its Effects on Population Health**

**(Cidacs PHC)**

| ***Opening and contextualization*** | |
| --- | --- |
| **Time** | **Session** |
| 09:00—09:30 | **Welcome and opening**   - About Cidacs (Mauricio Barreto) - **Who are we?** *"Flash" introductions* - Presentation of the agenda |
| 09:30—09:35 | **Why are we here? The perspective of Public Engagement with Science**  *Adalton dos Anjos – CIDACS/Fiocruz Bahia* |
| ***Topic 1***  ***A model to measure the effect of PHC on mortality in children under 5 years-of-age*** | |
| 09:35—09:45 | **Cidacs PHC: what we have already researched and our current research questions**  *Elzo Pereira Pinto Junior – Cidacs/Fiocruz* |
| 09:45 – 10:15 | **We want to learn with you:**   - Does the model of building blocks and clusters of services for maternal and child care in PHC that we developed adequately represent the reality of the resources available to PHC teams and the services that these teams provide? |
| ***Topic 2***  **Measuring PHC quality components across the three PMAQ cycles** | |
| 10:15 —10:25 | **Construction of PHC quality indicators**  *Maria del Pilar Flores-Quispe – Cidacs/Fiocruz Bahia* |
| 10:25—10:55 | **We want to learn with you:**   - Do the building blocks adequately reflect the planning processes of the PHC teams and the infrastructure of the PHC units to provide adequate child health care? - Do the components related to Prenatal Care, Child Health and Immunization adequately represent the actions carried out in maternal and child health care in PHC? |
| 10:55 – 11:10 | **Coffee/Tea break** |
| 11:10—11:20 | **Results: changes in quality indicators over the 3 PMAQ cycles**  *Maria del Pilar Flores-Quispe – Cidacs/Fiocruz Bahia* |
| 11:20—11:50 | **We want to learn with you:**   - Do the results of the evolution of these indicators between the three PMAQ cycles correspond to what you have observed in your daily routine? - Is this model useful for monitoring and evaluating the quality of maternal and child health care in the PHC? |
| ***Topic 3***  **An indicator to measure the quality of the workforce in PHC** | |
| 11:50—12:00 | **Presenting the variables of the National Registry of Health Establishments (CNES)**  *Laura de Almeida Botega – Cidacs/Fiocruz Bahia* |
| 12:00—12:30 | **We want to learn with you:**   - What information should be considered when building our workforce quality component for APS? |
| 12:30 —13:30 | **Official meeting photo & Lunch** |
| 13:30 – 14:00 | **Tour of Cidacs/Fiocruz Bahia** |
| ***Topic 4***  **Evolution of PHC financing and its effects on the quality of maternal and child health care** | |
| 14:00 - 14:10 | **Characterizing PHC financing in the period 2010-2017**  *Valentina Martufi – Cidacs/Fiocruz Bahia* |
| 14:10-14:40 | **We want to learn with you:**   - In terms of financing, what has a greater impact on the quality of Primary Health Care services: the amount of money available to the municipality (total revenue, including federal and state transfers and own resources), or how this money is spent (expenses - including revenue, commitments, settlements and payments)? - Considering revenue for PHC, is it worth studying federal transfers and own resources separately, or is the total amount available for the provision of PHC services more important? |
| 14:40-14:50 | **How funding for PHC affects the quality of the PHC building blocks**  *Valentina Martufi – Cidacs/Fiocruz Bahia* |
| 14:50-15:20 | **We want to learn with you:**   - What is the time lag that we should consider between financing (available or spent) and its effect on the quality of the structure available for PHC (building blocks)? - We observed an apparently pro-equity effect of the transfers of the Variable Primary Care Funding (PAB) (greater receipt of per capita resources in municipalities with greater material deprivation). Do you consider that this reflects the reality of PHC financing in Brazil? |
| 15:20-15:30 | **Coffee/Tea break** |
| ***Topic 5***  **Gender Dynamics and Health Services Management** | |
| 15:30-15:40 | **Construction of an indicator of adequacy of municipal management of health services**  *Elzo Pereira Pinto Junior – Cidacs/Fiocruz Bahia* |
| 15:40-16:10 | **We want to learn with you:**   - Considering the data available to us, what is your opinion on the adequacy indicator of municipal management of the health system? - In your opinion, what influenced the increase in the proportion of women in charge of municipal health secretariats? - What assumptions would you make to explain the findings related to the evolution in the adequacy of municipal management? |
| 16:10-16:20 | **Determinants of the adequacy of municipal management of the health system**  *Eduarda dos Anjos – Cidacs/Fiocruz Bahia* |
| 16:20-16:50 | **We want to learn with you:**   - What do you consider to be determinants for the adequacy of municipal health management? - How can we explain the result found that shows the association between municipal health management being led by a woman and greater adequacy in this indicator? |
| ***Closing*** | |
| 16:50-17:00 | - *Final considerations and next steps* |
