# Supplementary material for: Public engagement with science: an inclusive approach to innovate in health research with real-world data
Source: BMC Med Res Methodol. 2025 Apr 4;25:88. doi: 10.1186/s12874-025-02530-4 (PMC11970009; doi:10.1186/s12874-025-02530-4)
Supplement: Supplementary file 3 — Additional file 3. Agenda for the pes activity—Cidacs-PHDC Project. [file 12874_2025_2530_MOESM3_ESM.docx]

**ADDITIONAL FILE 3**

**AGENDA FOR THE PES ACTIVITY - CIDACS-PHDC PROJECT**

| 10.04.2024 | Session | Facilitators |
| --- | --- | --- |
| 9-9.30am | Welcome, presentation of members of the project | Adalton |
| 9.30-10.30am | Brazilian stakeholders experiences – Bahia Health Secretariat (Diego Cavalcanti), Ministry of Health (Blanda Mello) and National Health Secretaries Council (CONASS) (Felipe Ferre) | Adalton |
| 10.30-11.00am | Coffee Break |  |
| 11.00am-12.00pm | South African stakeholders experiences - MoH and Western Cape Intelligence Unit (Melvin Moodley - Director) | Valentina |
| 12.00-1.00pm | Lunch |  |
| 1.00-3.00pm | Presentation of the research project + Discussion about the best strategy regarding to CDM | Adalton |
| 3.00-3.30pm | Coffee break |  |
| 3.30-5.00pm | Integration activity with the group |  |
