# Supplementary material for: Public engagement with science: an inclusive approach to innovate in health research with real-world data
Source: BMC Med Res Methodol. 2025 Apr 4;25:88. doi: 10.1186/s12874-025-02530-4 (PMC11970009; doi:10.1186/s12874-025-02530-4)
Supplement: Supplementary file 4 — Additional file 4. [file 12874_2025_2530_MOESM4_ESM.pdf]

Engajamento Público com a Ciência: uma abordagem inclusiva para inovar na pesquisa em saúde com Dados do Mundo Real

Autores: Adalton dos Anjos Fonseca[1, 2], Valentina Martufi1, Walisson Angélico de Araújo[1], Denise Moraes Pimenta[1,3], Acácia Mayra Pereira de Lima[1,10], Juliana Araújo Prata de Faria[1], Danilo Luis Cerqueira Dias[1], Eduarda Ferreira dos Anjos[1], Maria del Pilar Flores Quispe[1], Gisela Rodrigues Piloto[4], Vivian Mitiko Queiroz Lima[4], Felipe Ferré[5], Marcos Antônio Gêmeos Almeida Sampaio[6], Erika de Souza Lopes[7], Blanda Helena de Mello[8], Diego Cavalcante Teixeira Daltro[9], Mariana Rodrigues Sebastião de Almeida[1], Raiza Tourinho Lima[1], Elzo Pereira Pinto Junior[1], Mauricio L. Barreto[1], Maria Yury Ichihara[1]

1 Centro de Integração de Dados e Conhecimentos para Saúde (CIDACS), Instituto Gonçalo Moniz, Fundação Oswaldo Cruz (FIOCRUZ);
2 Universidade Estadual do Sudoeste da Bahia (UESB), Salvador, Brasil
3 Universidade Federal de São Paulo, São Paulo, Brasil
4 Secretaria Municipal de Saúde de Salvador; Salvador, Brasil
5 Conselho Nacional de Secretários de Saúde (Conass); Brasília, Brasil
6 Conselho Estadual de Saúde da Bahia; Salvador, Brasil
7 Umane; São Paulo, Brasil
8 Ministério da Saúde; Brasília, Brasil
9 Secretaria Estadual de Saúde da Bahia; Salvador, Brasil
10 Universidade Federal do Paraná (UFPR), Curitiba, Brasil

*Autor correspondente: Adalton dos Anjos Fonseca adalton.anjos@gmail.com

Resumo

- **Contexto**

Engajamento Público com a Ciência (EPC) em pesquisas em saúde que utilizam big data para analisar desigualdades sociais em saúde exigem estratégias e métodos adaptados aos contextos dos países do Sul Global. Este artigo tem como objetivo examinar como, no Brasil – um país com forte tradição de participação social em pesquisa e formulação de políticas públicas – dois projetos de pesquisa de um centro que utiliza dados administrativos e do mundo real incorporam a inclusão e a diversidade como elementos-chave para conectar a produção de conhecimento com os desafios do mundo real.

- **Métodos**

O estudo analisa como dois projetos de pesquisa do Cidacs – um relacionado à Atenção Primária à Saúde (APS) e outro à Interoperabilidade de Dados – envolveram membros do público ao longo de sua implementação. Ambos os

projetos engajaram conjuntamente 18 participantes, incluindo representantes da comunidade, profissionais de saúde e gestores do setor público. Uma abordagem de relato de caso está sendo empregada neste artigo para documentar sistematicamente as experiências de EPC com base em um roteiro predefinido, abrangendo contexto, metodologia, atividades, públicos e aspectos éticos. Os dados foram coletados por meio de observação participante e escuta durante as atividades de engajamento, que facilitaram o diálogo entre participantes e pesquisadores, bem como por meio de questionários de acompanhamento e discussões subsequentes. Este próprio artigo surgiu desse processo colaborativo, incluindo alguns participantes do EPC como coautores.

- **Resultados**

A colaboração dos participantes impactou as decisões dos pesquisadores, proporcionando uma compreensão mais próxima dos desafios enfrentados pelos participantes em seu trabalho diário em relação a cada um dos temas de pesquisa. Além disso, essas discussões resultaram no estabelecimento de parcerias para novas iniciativas. Os participantes destacaram que, além da oportunidade de contribuir para o desenvolvimento de pesquisas científicas, adquiriram novos conhecimentos a partir do contato com as equipes de pesquisa.

- **Conclusões:**

A diversidade de grupos sociais e a inclusão de diferentes perspectivas em projetos de pesquisa mobilizados pelo EPC têm o potencial de promover inovações nos processos e resultados da pesquisa, além de gerar impacto social. O potencial de aplicabilidade das informações científicas é ampliado, uma vez que estão mais conectadas ao mundo real, e os próprios participantes impulsionam o processo de disseminação.

1. **Contexto**

As publicações sobre Engajamento Público com a Ciência (EPC) em pesquisas em saúde cresceram significativamente. Na plataforma PubMed, o número de artigos aumentou de 77 para 258 entre 2013 e 2023[1]. As iniciativas variam desde ações em que os participantes influenciam decisões em saúde[1,2], sugerem melhorias nos serviços de saúde[3] ou participam de pesquisas que aplicam metodologias participativas, como em Rubio et al.[4]

O EPC refere-se a um dos níveis de participação de diferentes segmentos sociais no processo de construção do conhecimento científico[5]. Inicialmente desenvolvido em países do Norte Global, é mais conhecido no Brasil como participação social. Está profundamente ligado às lutas por direitos humanos e alinhado com movimentos sociais. Um bom exemplo disso foi a criação e construção do Sistema Único de Saúde (SUS). O SUS foi criado em 1988 e visava reduzir as iniquidades em saúde no país, garantindo acesso universal, integral e gratuito aos serviços de saúde para todos[6,7].

Alguns dos atores que participaram das lutas sociais pela criação do SUS, da academia e da gestão pública, fundaram o Centro de Integração de Dados e Conhecimentos para Saúde (Cidacs/Fiocruz Bahia) em 2016. O centro de pesquisa foi criado para realizar estudos multidisciplinares que utilizam a vinculação de grandes bancos de dados administrativos para gerar evidências sobre os determinantes sociais da saúde e avaliar o impacto de políticas públicas na saúde da população, utilizando Dados do Mundo Real (DMR)[8]. Uma conquista notável do Cidacs foi a criação de duas grandes coortes utilizando exclusivamente bancos de dados administrativos vinculados[9,10].

Este artigo apresenta uma abordagem metodológica para o EPC baseada em estratégias que permitem a inclusão e a diversidade em pesquisas em saúde realizadas com DMR. Utilizando duas experiências de projetos de pesquisa do Cidacs relacionados à Atenção Primária à Saúde (Cidacs APS) e à interoperabilidade de dados (Cidacs-PHDC), apresentaremos os desafios e soluções desenvolvidos, buscando contribuir para a sistematização de uma estratégia em processos de pesquisa que promovam a inclusão e a diversidade na produção de conhecimento. Também demonstraremos os impactos e lições aprendidas com essas iniciativas.

2. Definindo engajamento público com a ciência

O Engajamento Público com a Ciência (EPC) refere-se à inclusão, no processo de produção de conhecimento, de vozes comumente marginalizadas nos debates públicos e não diretamente envolvidas em atividades profissionais relacionadas às disciplinas científicas[5,11,12]. Também se estende à participação de formuladores de políticas, comunidades profissionais, legisladores, ONGs e instituições intergovernamentais como uma estratégia para consultar, colaborar e cocriar conhecimentos úteis que informem a tomada de decisões coletivas[11].

A Declaração de Helsinque da Associação Médica Mundial[13] enfatiza a importância de envolver participantes e suas comunidades antes, durante e após a pesquisa. Enquanto isso, Arnstein[14] destaca a necessidade de uma redistribuição de poder ao longo do processo de participação, defendendo uma reestruturação fundamental das dinâmicas de tomada de decisão e da relação entre ciência e sociedade. O Espectro de Participação Pública é um modelo útil da Associação Internacional de Participação Pública (IAP2) que categoriza os níveis de envolvimento dos cidadãos na tomada de decisões com base em cinco modos de participação – informar, consultar, envolver, colaborar e empoderar.

Pesquisadores no Reino Unido e nos Estados Unidos moldaram esse conceito dentro da comunidade científica. No entanto, ao longo do tempo, ele foi adotado em uma escala mais ampla[5,12]. Na América Latina, várias adaptações foram implementadas devido aos contextos sociais, políticos e culturais únicos da região, e sua forte tradição de participação social. Iniciativas revolucionárias de ativistas brasileiros em educação popular e saúde pública, como as de Paulo Freire[15,16] e Sergio Arouca[17], foram fundamentais para a construção de uma sociedade amplamente engajada na luta por direitos. As iniciativas na região são guiadas por diversos princípios e valores. No entanto, o empoderamento é um tema central, com grupos comunitários influenciando ativamente as decisões em saúde e ganhando capacidade de fazer escolhas informadas. A diversidade é importante para garantir uma variedade de pontos de vista. A inclusão e a colaboração entre pesquisadores, profissionais de saúde, formuladores de políticas e comunidades são enfatizadas nessas atividades que abordam desafios do mundo real[18,19].

No Cidacs, o EPC concentra-se no compartilhamento de experiências e conhecimentos entre pesquisadores e partes interessadas do sistema de saúde para gerar evidências que apoiem políticas sociais. O trabalho realizado vai além de grupos comunitários – por exemplo, populações tradicionais ou moradores de favelas –, uma vez que há colaborações com gestores públicos, formuladores de políticas e organizações intergovernamentais (como a Organização Mundial da Saúde e as Nações Unidas), grupos de advocacy e entidades civis, trabalhadores da saúde, profissionais de mídia e educação, além de acadêmicos especializados nos temas estudados. O envolvimento dessas partes interessadas, os *stakeholders,* é cuidadosamente planejado e implementado progressivamente, considerando as demandas e necessidades específicas. Os resultados dessa

colaboração têm sido significativos para os projetos de pesquisa, tornando-os mais alinhados com o mundo real e aumentando seu potencial de aplicabilidade.

No âmbito deste trabalho, a equipe do Cidacs desenvolve uma série de tarefas, desde a pesquisa até a avaliação, de acordo com o fluxo de trabalho abaixo (Figura 1).

Figura 1 - Fluxo de trabalho das atividades de engajamento público com a ciência no Cidacs

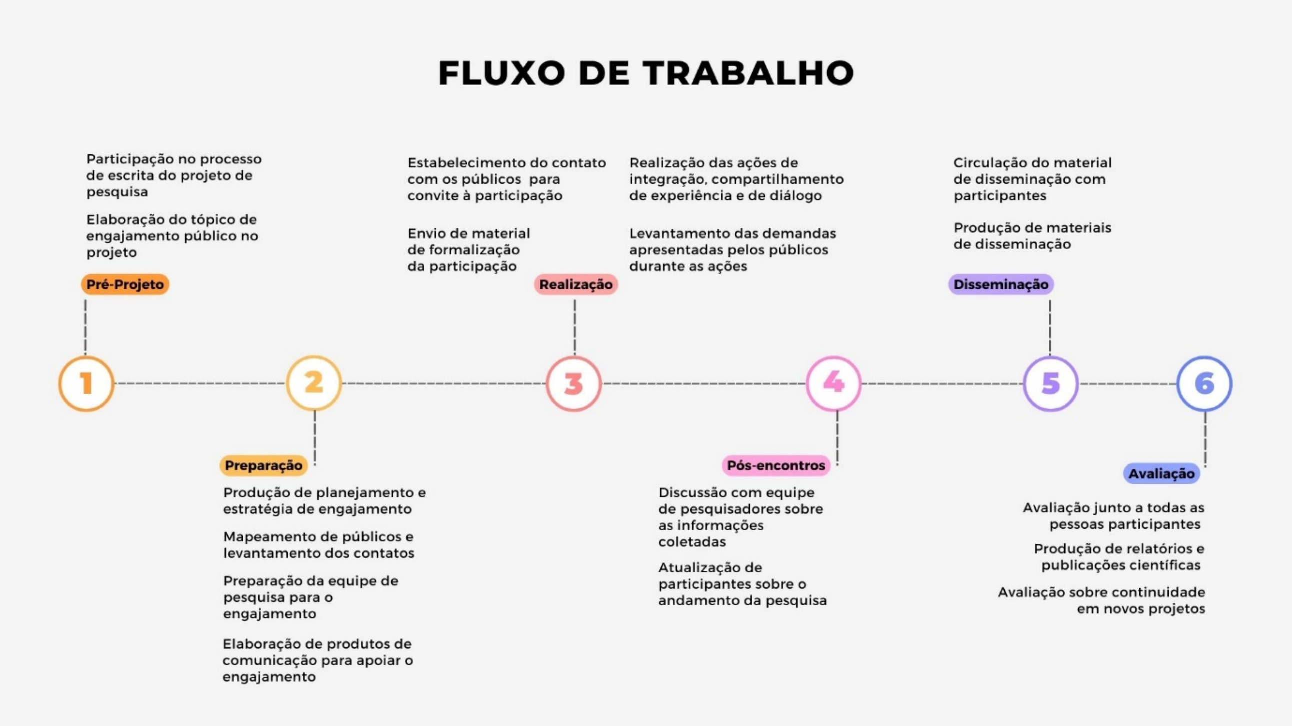

Fonte: elaboração própria dos autores

3. **Métodos**

Os casos apresentados envolvem dois projetos de pesquisa realizados no Cidacs, incluindo diferentes públicos – uma instituição representativa da comunidade, profissionais de saúde e técnicos de gestão em saúde – desde o início do processo de pesquisa. Desde a concepção de cada projeto de pesquisa, os pesquisadores indicaram a intenção de realizar EPC, adaptando as diretrizes da seção 6 da Declaração de Helsinque de 2024 da Associação Médica Mundial ao contexto brasileiro. Os procedimentos para seleção e construção de relacionamentos com os participantes nas atividades de engajamento público de ambos os projetos seguiram estas etapas principais:

1. Mapeamento de instituições relevantes em níveis local, estadual e nacional relacionadas aos principais temas dos projetos;
2. Validação da lista de instituições com pesquisadores sêniores do projeto e cinco partes interessadas de outras iniciativas de pesquisa no Cidacs por meio de conversas individuais;
3. Identificação de participantes dentro dessas instituições para acompanhar o projeto;
4. Envio de convites formais explicando a iniciativa;
5. Estabelecimento de uma relação de confiança com as partes interessadas por meio de atualizações periódicas sobre os projetos.

Um total de 18 indivíduos participaram das atividades dos dois projetos, de 27 convidados a participar do grupo. Entre eles, 11 eram gestores do setor público, quatro eram profissionais de saúde, um representava organizações da sociedade civil, um representava grupos comunitários e um era da comunidade acadêmica. Seus papéis variavam de coordenadores ou assessores no setor de APS, médicos de família, enfermeiros ou membros de conselhos de saúde. A maioria dos integrantes eram mulheres (13) e representantes de instituições locais e estaduais (13). Além disso, cinco participantes representavam instituições federais.

As atividades de engajamento incluíram reuniões técnicas presenciais e online, onde pesquisadores e participantes discutiram questões de pesquisa, metodologias, fontes de dados, ferramentas analíticas e interpretações de resultados, conforme detalhado nas seções seguintes deste artigo. Os dados foram coletados por meio de observação participante durante as atividades de engajamento e por meio de questionários estruturados e formulários de feedback abertos.

Para apresentar as iniciativas de interesse, adotamos o relato de caso – uma forma de produção de conhecimento baseada na reflexão crítica a partir de uma experiência acadêmica ou profissional. Com base em um roteiro[20], sistematizamos as experiências inclusivas de EPC com base em sua contextualização, os procedimentos metodológicos adotados para cada intervenção e a caracterização dos projetos de pesquisa em termos de tempo, espaço, atividades, públicos e aspectos éticos.

3.1. Iniciativas de EPC para os projetos CIDACS APS e CIDACS-PHDC

As iniciativas relatadas são baseadas no mesmo modelo para estruturar os procedimentos e valores envolvidos nas atividades de EPC, embora alguns ajustes sejam feitos para atender às necessidades específicas de cada projeto. Para configurar as atividades de engajamento para os projetos descritos abaixo, pesquisadores associados aos projetos, juntamente com a equipe de engajamento – composta por pesquisadores de comunicação e antropologia – realizaram reuniões para:

1) Mapear potenciais participantes, incentivando a representatividade de acordo com raça, gênero, faixa etária, região e diferentes funções/cargos;

2) Desenvolver estratégias de recrutamento (uso de e-mails institucionais, mensagens instantâneas, telefone e mídias sociais para enviar convites com uma mensagem sobre o projeto e o workshop, bem como o compartilhamento gradual de materiais complementares/informativos sobre o projeto);

3) Organizar roteiros de workshops usando linguagem acessível, incluindo perguntas orientadoras, e treinar a equipe de pesquisa para dar espaço e ouvir efetivamente as experiências das partes interessadas;

4) Desenvolver um modelo de avaliação para as atividades e resultados obtidos.

Todas as atividades de engajamento são apoiadas pelo grupo de comunicação e disseminação científica do Cidacs, que garante níveis de engajamento por meio de consulta, envolvimento e colaboração.

3.1.1. Cidacs APS

O grupo Cidacs APS é dedicado ao estudo do impacto da Atenção Primária à Saúde (APS) na saúde da população. Na primeira fase do projeto, o efeito da qualidade da APS na mortalidade infantil foi explorado. Em sua segunda fase, com uma estratégia estruturada de EPC, o foco tem sido aprofundar os achados da fase anterior, incluindo questões de pesquisa sobre os determinantes da qualidade da APS. A primeira atividade de engajamento foi um workshop na sede do Cidacs em Salvador (Brasil), onde quinze pessoas, incluindo gestores de saúde pública, trabalhadores da saúde, pesquisadores de outras instituições e representantes da sociedade civil e de grupos comunitários, discutiram os

resultados da primeira fase do projeto e as questões de pesquisa da segunda fase. Esta primeira atividade introdutória de engajamento foi presencial e durou um dia inteiro, o que permitiu a cobertura extensiva de todas as questões de pesquisa deste projeto. Posteriormente, três reuniões de acompanhamento foram focadas em questões de pesquisa específicas, realizadas online e com duração mais curta (90 minutos), devido a restrições logísticas e orçamentárias. Estas representaram a continuação do processo de engajamento, à medida que o grupo de pesquisa avançava com as análises e trazia seus achados de volta ao comitê de engajamento para discussão e tomada de decisão conjunta em relação aos próximos passos.

3.1.2. Projeto Cidacs-PHDC

Há uma colaboração entre o Cidacs e o Western Cape Provincial Health Data Centre (PHDC) na África do Sul que visa criar um Modelo Comum de Dados (CDM) para estudar doenças infecciosas que afetam a gravidez, como sífilis gestacional e tuberculose, utilizando dados do Brasil e da Província do Cabo Ocidental. A criação de um CDM visa estruturar os bancos de dados de cada instituição de acordo com um padrão unificado de tabelas, campos e relações projetados para harmonizar dados de diferentes fontes. Para isso, utiliza vocabulário controlado, conceitos e códigos, garantindo uma interpretação consistente dos dados, independentemente de sua origem.

Uma atividade de engajamento com especialistas em Big Data e gestores de saúde de órgãos governamentais nacionais e locais foi realizada para discutir o desenvolvimento e a aplicação do CDM nos contextos brasileiro e sul-africano. Durante a sessão de engajamento, os participantes trocaram experiências e discutiram tópicos relacionados à utilização de um modelo comum de dados que considere as especificidades sociais e econômicas do Sul Global. Essas discussões foram fundamentais no desenvolvimento de um CDM que promova a padronização de dados a partir de uma perspectiva inclusiva e colaborativa.

O processo de tomada de decisão em relação à adoção do CDM pelos governos influenciará a disponibilidade de dados de boa qualidade para que os pesquisadores realizem análises significativas que possam informar os formuladores de políticas e melhorar os sistemas de saúde. Três representantes da administração pública do Brasil e dois representantes do Departamento de Saúde da Província do Cabo Ocidental da África do Sul participaram da primeira atividade de EPC para este projeto. O principal produto desta iniciativa é um

documento de recomendações sobre o CDM para as partes interessadas – profissionais, formuladores de políticas e outras instituições.

3.2. Atividades com os participantes

As atividades de engajamento para ambos os projetos incluíram uma combinação de reuniões presenciais e online, promovendo um diálogo significativo entre pesquisadores e participantes. No projeto Cidacs-APS, os participantes foram convidados a revisar criticamente, expandir e refinar questões de pesquisa, fontes de dados, abordagens analíticas e interpretações de resultados. As apresentações dos pesquisadores foram seguidas por discussões focadas, onde os participantes puderam compartilhar sugestões, críticas e perguntas, criando um ambiente colaborativo e inclusivo. Para garantir transparência e engajamento contínuo, todos os materiais de apresentação foram compartilhados com os participantes após as sessões, permitindo reflexão e contribuições adicionais.

No projeto Cidacs - PHDC, participantes e pesquisadores apresentaram soluções e experiências sobre interoperabilidade de dados de seus respectivos locais de trabalho, destacando a diversidade de abordagens em diferentes contextos. Após as apresentações, um fórum de discussão foi estabelecido para facilitar a troca de ideias e possíveis colaborações. Essa abordagem inclusiva permitiu que os participantes contribuíssem para os objetivos, metodologias e resultados finais do projeto de pesquisa, orientando novas direções e possibilidades para trabalhos futuros.

Além disso, é importante destacar que a produção deste próprio artigo é um resultado dessa estratégia de cooperação inclusiva, já que alguns participantes também são coautores. Todos os participantes foram convidados a contribuir por meio de um e-mail contendo cinco perguntas abertas, com a opção de responder por meio de um documento anexado ou de um formulário online. No total, 18 indivíduos foram convidados, e 7 concordaram em participar, enviando contribuições que refletiram suas perspectivas sobre as atividades de engajamento e revisando o manuscrito.

4. **Resultados**

4.1. Processo de engajamento

Durante as reuniões (duas presenciais e três online), questões foram abordadas a partir de diferentes perspectivas e realidades, resultando em uma experiência rica de compartilhamento de diferentes abordagens para resolver problemas complexos. Os participantes apreciaram a oportunidade de aprendizado recíproco e troca de conhecimentos com profissionais de diferentes níveis de *expertise*. Eles também apontaram que esse modelo de pesquisa baseado na inclusão de ideias de diversas pessoas permite que a ciência esteja mais próxima do território e alcance resultados mais relevantes, inclusivos e representativos.

4.2. Impacto nos projetos de pesquisa

Para a equipe do Cidacs APS, a reunião com os participantes proporcionou cinco principais *insights*: a) a necessidade de incluir urgentemente questões raciais no escopo das análises; b) a importância de identificar outras variáveis para avaliar a qualidade da APS, como o tipo de contrato de trabalho e a sobrecarga de trabalho para representar a qualidade da força de trabalho; c) o potencial impacto do perfil profissional e do gênero do Secretário de Saúde na adequação da gestão municipal; d) o contexto e as nuances relacionadas ao período de tempo da pesquisa; e, e) o acordo sobre tópicos importantes, como o tempo de defasagem entre o recebimento de recursos financeiros e a manifestação de melhorias na qualidade da APS.

Para o projeto Cidacs-PHDC, a atividade permitiu a validação da importância desse movimento para construir capacidade de interoperabilidade de dados e a aplicação de um CDM, para promover o uso de dados do mundo real. Todos os participantes reconheceram que um CDM específico, desenvolvido pelo OMOP (Observational Medical Outcomes Partnership), é um caminho válido, do ponto de vista da ciência aberta, a ser explorado para romper fronteiras no conhecimento em saúde[2]. No entanto, os gestores de dados que participaram das discussões indicaram que o CDM OMOP especificamente é mais útil para pesquisa e produção de conhecimento em saúde, mas não é particularmente útil para a gestão de dados de saúde por entidades estaduais. De fato, em ambos os

países, outros CDMs já estão sendo testados com foco no uso primário de dados de saúde para apoiar a prestação de serviços.

Nesse sentido, a Estratégia de Saúde Digital adotada no Brasil estabelece governança para a troca de informações com modelos acordados pela Comissão Intergestores Tripartite (CIT). Os participantes observaram que a reconciliação entre soluções acadêmicas e estratégias de gestão poderia acelerar a descoberta de conhecimento, reduzir o tempo de resposta translacional e, simultaneamente, oferecer suporte oportuno para a tomada de decisões para cidadãos, profissionais e gestores. Eles sugeriram novas partes interessadas e grupos comunitários para incluir nessa discussão, bem como algumas estratégias de advocacy.

3. **Avaliação contínua**

O processo de avaliação contínua, que avalia o nível de interesse e engajamento dos participantes, envolve a análise de sugestões, críticas e comentários compartilhados durante e após as reuniões. Depoimentos informais e avaliações em plenária também serviram como fontes valiosas de *insights*. Além disso, um questionário de avaliação pós-atividade foi aplicado, oferecendo uma abordagem estruturada para avaliar a satisfação e a confiança dos participantes no processo adotado. As respostas foram 100% positivas, com as quatro perguntas fechadas abordando aspectos-chave, como a qualidade geral da reunião, a metodologia, a clareza das apresentações, o interesse em continuar participando, os destaques e sugestões de melhoria. No entanto, um desafio surgiu nas respostas descritivas: a complexidade dos tópicos do projeto muitas vezes excedeu o tempo alocado para discussão, que foi limitado a 40 minutos a 1 hora por tópico. Apesar disso, o envolvimento sustentado dos participantes em atividades de engajamento subsequentes (taxa de retorno superior a 90%), bem como a coautoria deste manuscrito por alguns participantes, reforçam o estabelecimento de uma relação construída com base na confiança.

5. Discussão

A operacionalização de conceitos-chave como diversidade e inclusão são tarefas complexas. O desenvolvimento e a implementação desses processos envolvem discussões principalmente em torno de raça e gênero, que são fatores estruturais nos processos de construção de desigualdades em nossa região[2] , mas também se intersectam com outras categorias, como status socioeconômico, idade,

orientação sexual, crenças religiosas, ideologias, habilidades físicas, entre outras. Discutir essas questões exige considerações sobre como, ao longo da história, teorias e metodologias do Norte Global foram importadas para o Sul Global sem problematizar seu impacto epistemológico e tecnocientífico[2].

Desenvolver um modelo comum de dados para pesquisa em saúde sem incorporar as especificidades contextuais das populações em países com profundas desigualdades sociais destaca um desafio significativo. A produção de conhecimento, muitas vezes impulsionada pelo Norte Global na busca pela padronização internacional de dados, deve incluir ativamente perspectivas diversas que só podem ser plenamente compreendidas por meio da prática no mundo real. A falha em considerar essas características contextuais corre o risco de criar modelos de dados desalinhados com as realidades das populações vulneráveis, limitando sua representatividade e eficácia. Os resultados de tal abordagem influenciarão significativamente como essas populações são analisadas e monitoradas, impactando diretamente o desenvolvimento de políticas públicas.

As atividades de EPC relatadas neste artigo apresentaram resultados importantes devido aos esforços para atender à diversidade de perfis de grupos sociais e incluir diferentes perspectivas em projetos de pesquisa. Além de gênero e raça como critérios de inclusão, outros aspectos importantes para construir uma diversidade de perfis foram considerados para o projeto Cidacs APS, como função de trabalho, formação acadêmica, experiência profissional, nível hierárquico e instituição de origem. A atividade do Cidacs-PHDC, por outro lado, envolveu um número menor de participantes devido à complexidade e especificidade do tópico discutido – interoperabilidade de dados e CDMs. No entanto, foi feito um esforço para representar diferentes instituições governamentais para uma discussão mais rica. Ainda assim, a oportunidade foi aproveitada para explorar o mapeamento de públicos-alvo para maior diversidade.

Compreender o contexto regional, com foco em estratégias inclusivas e diversas que promovam a cooperação entre pesquisadores e sociedade, é essencial para intervenções relacionadas ao processo de engajamento[23]. Nesse sentido, algumas lições de Reynolds e Sariola[24] permearam nossas decisões, manifestando-se em processos de engajamento como a escuta ativa das experiências cotidianas dos participantes, o impacto das contribuições dos

participantes nos processos de pesquisa e o estabelecimento de novas parcerias e colaborações.

Esse processo de inclusão também envolve algumas barreiras que limitam as estratégias adotadas. O tempo foi uma das principais barreiras indicadas por todos os participantes. Além disso, a equipe observou que a inclusão de pessoas de diferentes níveis hierárquicos na atividade do projeto Cidacs APS, embora importante para acessar diferentes pontos de vista, resultou em restrições na expressão de ideias divergentes devido a desequilíbrios de poder entre os participantes. No caso do projeto Cidacs-PHDC, os diferentes contextos e idiomas também foram aspectos que precisaram ser observados no processo de EPC. Além disso, a implementação de algumas sugestões dos participantes foi difícil devido à falta de variáveis específicas nos bancos de dados disponíveis. No entanto, isso também representou uma oportunidade para refletir sobre novas fontes de dados que poderiam ser utilizadas, bem como enriquecer o processo dos pesquisadores de reconhecer ativamente as limitações de seu trabalho. Também reconhecemos o potencial de viés no mapeamento de participantes de ambos os projetos; no entanto, essa abordagem foi escolhida principalmente devido a considerações de custos de viagem e à rápida mobilização de participantes.

As lições aprendidas incluem a importância do planejamento detalhado de cada etapa do trabalho de engajamento, desde o objetivo e o mapeamento das partes interessadas até o agendamento de cada sessão, seguindo as recomendações de dos Anjos Fonseca[5]. A formulação de perguntas orientadoras para o debate entre pesquisadores e participantes mostrou-se muito benéfica, pois mobilizou os participantes e estimulou as discussões.

Outro aspecto importante dessa relação construída entre pesquisadores e participantes é a dimensão ética do engajamento público. Dimensões éticas críticas que compõem o quadro para processos de participação e ciência cidadã[25] foram contempladas, como empatia e a construção de espaços comunicativos, o levantamento de questões que permeiam aspectos éticos – como ética de dados e questões de raça e gênero –, a promoção do diálogo e o fomento de relacionamentos sem preconceitos. Nesse sentido, a comunicação é fundamental para estruturar uma relação ética e colaborativa entre pesquisadores e participantes do ETC. A inclusão de pesquisadores de comunicação e antropologia na equipe foi importante porque enfatizou a importância da

transparência, conscientizando os pesquisadores sobre a entrega de apresentações com uma linguagem acessível e promovendo a escuta ativa e o acordo prévio sobre o programa entre participantes e pesquisadores. Isso foi feito por meio de reuniões, mensagens estratégicas enviadas aos participantes antes e após as atividades, conversas informais e processos de avaliação durante as reuniões.

Figura 2 - Lições aprendidas e impactos do EPC em ambas as iniciativas

**Lições Aprendidas**	**Impactos**
Considerar contextos locais e regionais (ex.: geográfico, histórico, político e cultural) quando selecionar os participantes	A diversidade de contribuições baseadas em seus contextos específicos enriquecerá o processo de pesquisa.
Planeje cada etapa do processo de EPC com cuidado.	As reuniões serão mais produtivas e atingirão efetivamente seus objetivos.
Estabelecer acordos prévios sobre o programa entre participantes e pesquisadores.	Incentiva a participação ativa em todo o processo e prepara os participantes para as discussões.
Use perguntas orientadoras para facilitar o diálogo.	Ajuda a estruturar conversas e incentiva a participação ativa.
Pratique a escuta ativa e respeitosa de todas as sugestões e comentários sem preconceitos.	Constrói confiança e promove a participação aberta e destemida.
Inclua pesquisadores de comunicação, antropólogos ou especialistas em comunicação pública na equipe.	Permite o desenvolvimento de estratégias robustas de EPC e alcança resultados complexos.
Use linguagem simples e seja transparente, especialmente sobre as limitações do seu trabalho.	Facilita o diálogo claro e permite que os participantes forneçam sugestões ou feedback mais precisos.
Mantenha uma comunicação contínua com os participantes.	Fortalece as relações para o projeto atual e promove a colaboração em iniciativas futuras.
Monte uma equipe diversificada e fornecer treinamento em EPC e comunicação pública.	Melhora a comunicação entre pesquisadores e participantes, maximizando os resultados do projeto.

Fonte: elaboração própria dos autores

Como esta intervenção demonstra na prática, uma barreira a ser superada é que a ciência deve ser produzida não apenas para a sociedade, mas com a sociedade. Ao considerar a diversidade e ir além da fronteira acadêmica com esse tipo de intervenção na área da saúde, o Cidacs integra conhecimento e subverte o padrão das elites "decidindo por" as massas. Esse movimento também é essencial para a democratização do conhecimento e para o aumento da confiança nas instituições de pesquisa, sua produção e recomendações. Além de expandir a compreensão dos dados, as atividades de EPC fornecem informações contextuais adicionais que enriquecem a pesquisa com base em achados da análise de extensos bancos de dados de saúde, conectando os achados às realidades locais e aos desafios e necessidades concretas da população.

Os próximos passos para essas iniciativas incluem a continuação das atividades de engajamento para discutir as direções futuras dos projetos de pesquisa. Isso envolverá a expansão do grupo que supervisiona o projeto Cidacs-PHDC para incluir representantes de grupos comunitários e organizações da sociedade civil, garantindo diversidade na representação por meio de critérios cuidadosamente definidos. Além disso, os participantes serão consultados e envolvidos na elaboração de estratégias para divulgação científica e advocacy.

5. Conclusões

As experiências relatadas neste manuscrito destacam o papel estratégico do engajamento público com a ciência e da comunicação na execução de processos que ajudam a construir e fortalecer a articulação da produção de conhecimento com a realidade cotidiana. Isso permite que a comunidade científica dialogue com diversos segmentos sociais que trabalham diretamente com os temas de pesquisa de interesse.

Embora os projetos ainda estejam em andamento, é evidente que a combinação de EPC, comunicação e interdisciplinaridade destaca o papel crítico da escuta de qualidade e do diálogo significativo na identificação de pontos comuns e no fomento de inovações que ajudam a mitigar as desigualdades em saúde. Alcançar isso exige abordagens de pesquisa mais inclusivas que abracem perspectivas diversas e adaptem metodologias para atender melhor às necessidades específicas das populações. Também esperamos impactos na influência de especialistas em dados, formuladores de políticas e pesquisadores, bem como no

desenvolvimento de novas iniciativas que promovam a diversidade nas equipes de pesquisa.

Relatar essas experiências de cooperação e diálogo, além da análise de dados e discussões teóricas, nos permite compreender a importância de humanizar os serviços de saúde e a ciência. Expandir repertórios por meio do diálogo e da escuta provou ser essencial para implementar práticas mais inclusivas que vão além de indicadores quantitativos, valorizando contextos específicos e promovendo conexões significativas.

Reconhecer que o acesso e as capacidades são desiguais – tanto na ciência quanto no cuidado em saúde – é um passo fundamental para evitar a perpetuação de desigualdades. Métodos científicos muitas vezes falham em considerar as especificidades dos territórios e das populações que os habitam. Recomendamos a construção de capacidades em comunicação pública da ciência e EPC para aplicar metodologias participativas nessas práticas. Além disso, é importante ir além dos dados e números ao realizar pesquisas em saúde populacional. Ao ouvir e desenvolver diálogos e conexões inclusivas com diferentes partes interessadas e abrir mão de certezas pré-estabelecidas para expandir a capacidade de acolher outras perspectivas, pode ser possível implementar inovações e contribuir para um futuro mais equitativo sem repetir erros do passado.

Referências

[1] Ciapponi, A., Bardach, A., Alcaraz, A., Belizán, M., Jones, D., Comolli, M., & Ruvinsky, S. D. (2019). Taller de priorización de intervenciones para el control del mosquito Aedes aegypti en Latinoamérica y el Caribe: diálogo de políticas. [*Workshop for prioritizing interventions for the control of the Aedes aegypti mosquito in Latin America and the Caribbean: policy dialogue*.] *Cadernos de Saúde Pública*, *35*, e00092918.

[2] Velásquez-Jiménez, C. M., Sarmiento, M. I., Vargas, S., Fandiño, V., Puerto-de-Amaya, M. B., Ardila-Sierra, A., & Quintana, E. (2022). Estrategia educativa intercultural para la prevención del cáncer de cuello cérvico uterino en el resguardo de Paujil, Amazonia Colombiana. [*Intercultural educational strategy for the prevention of uterine cervical cancer in the Paujil shelter, Colombian Amazon*.] *Revista UDCA Actualidad & Divulgación Científica*, *25*(SPE).

[3] Frech, S., Muha, C. A., Stevens, L. M., Trimble, E. L., Brew, R., Perin, D. P., ... & Kostelecky, B. (2018). Perspectives on strengthening cancer research and control in Latin America through partnerships and diplomacy: experience of the national cancer institute's center for global health. *Journal of Global Oncology*, *4*, 1-11.

[4] Rubio, M. A., Guevara-Aladino, P., Urbano, M., Cabas, S., Mejia-Arbelaez, C., Rodriguez Espinosa, P., ... & Sarmiento, O. L. (2022). Innovative participatory evaluation methodologies to assess and sustain multilevel impacts of two community-based physical activity programs for women in Colombia. *BMC public health*, *22*(1), 771.

[5] dos Anjos Fonseca, A., Moraes Pimenta, D., Rodrigues Sebastião de Almeida, M., Tourinho Lima, R., Barreto, M. L. and Travassos Ichihara, M. Y. (2023). Public Involvement & Engagement in health inequalities research on COVID-19 pandemic: a case study of CIDACS/FIOCRUZ BAHIA. *International Journal of Population Data Science*. 5(3). doi: 10.23889/ijpds.v5i3.2133.

[6] Paim, J.S. O que é o SUS? [*What is the SUS?*] Rio de Janeiro: Editora Fiocruz, 2009.

[7] Paim, J. S. (2018). Sistema Único de Saúde (SUS) aos 30 anos. [*30 years of the Unified Health System.*] *Ciência & Saúde Coletiva*, *23*(6), 1723-1728.

8. Barreto, M. L., Ichihara, M. Y., Almeida, B. D. A., Barreto, M. E., Cabral, L., Fiaccone, R. L., ... & Smeeth, L. (2019). The centre for data and knowledge integration for health (CIDACS): linking health and social data in Brazil. *International Journal of Population Data Science, 4(2).*

9. Barreto ML, Ichihara MY, Pescarini JM, Ali MS, Borges GL, Fiaccone RL, Smeeth L. Cohort profile: The 100 million Brazilian cohort. *Int J Epidemiol*. 2022;51(2):e27–38. https://doi.org/10.1093/ije/dyab213

10. Paixão ES, Cardim LL, Falcão IR, Ortelan N, Silva NDJ, Rocha ADS, Teixeira MG. Cohort profile: Centro de Integração de Dados e Conhecimentos para Saúde (CIDACS) birth cohort. I*nt J Epidemiol*. 2021;50(1):37–8. https://doi.org/10.1093/ije/dyaa255

11. Sales CP. *Engajamento Comunitário e Entendimento Público da Ciência: primeiras anotações para uma agenda de pesquisa* [dissertação]. Rio de Janeiro: Fundação Oswaldo Cruz, Curso de Informação e Comunicação em Saúde, Instituto de Comunicação e Informação Científica e Tecnológica em Saúde; 2014. Disponível em: https://www.arca.fiocruz.br/handle/icict/11821

12. Weingart P, Joubert M, Connoway K. Public engagement with science—Origins, motives and impact in academic literature and science policy. *PLoS ONE*; 2021; 16(7): e0254201. https://doi.org/10.1371/journal.pone.0254201

13. World Medical Association. (2024). World Medical Association Declaration of Helsinki. *Ethical principles for medical research involving human subjects.* https://www.wma.net/what-we-do/medical-ethics/declaration-of-helsinki/

14. Arnstein, Sherry R. A ladder of citizen participation. *Journal of the American Institute of planners*, v. 35, n. 4, p. 216–224, 1969.

15. Freire P. *Educação e mudança.* 38ª. Rio de Janeiro/São Paulo: Paz e Terra; 2018.

16. Freire P. *Pedagogia do Oprimido.* 66ª. Rio de Janeiro/São Paulo: Paz e Terra; 2018.

17. Arouca, S. (2003). *O dilema preventivista: contribuição para a compreensão e crítica da medicina preventiva.* Rio de Janeiro: Editora Fiocruz; São Paulo: Editora da Unesp. E-book. https://doi.org/10.7476/9788575416105

18. Arias López, B. E. (2016). Saberes locales campesinos sobre el alimento: aportes a la soberanía y la salud mental comunitaria.

19. Presotto RF, Silveira M, Delgado PGG, Vasconcelos EM. Experiências brasileiras sobre participação de usuários e familiares na pesquisa em saúde mental. *Cien Saude Colet*. 2013;18:2837–45.

20. Mussi, R. F. F., Flores, F. F., Almeida, C. B. (2021). Pressupostos para a elaboração de relato de experiência como conhecimento científico. *Revista práxis educacional,* v.17, n. 48, p. 60–77.

21. Gonzalez, L. Por um Feminismo Afro-Latino-Americano. In: Gonzalez, L. (2018) *Primavera para as Rosas Negras*. São Paulo: UCPA Editora.

22. Fortes, C. Teorias que servem e teorias que não servem: dinâmicas familiares e de género em Cabo Verde e os desafios da importação teórica. In: Lobo, A., Dias, J. B. (Orgs.). *Mundos em circulação: perspectivas sobre Cabo Verde*. Brasília: ABA Publicações; LetrasLivres / Cidade da Praia: Edições Uni-CV, 2016.

23. Graham PW, Kim MM, Clinton-Sherrod AM, Yaros A, Richmond AN, Jackson M, Corbie-Smith G. What is the role of culture, diversity, and community engagement in transdisciplinary translational science? *Transl Behav Med.* 2016;6(1):115–24.

24. Reynolds L, Sariola S. The ethics and politics of community engagement in global health research. *Crit Public Health.* 2018;28(3):257–68.

25. Groot, B., Abma, T. (2022). Ethics framework for citizen science and public and patient participation in research. *BMC Med Ethics 23.* https://doi.org/10.1186/s12910-022-00761-4
